# Between Pressure and Coping: Stress Management Strategies and Social Support as Predictors of Abnormal Eating Behaviours in Young Polish Judo Athletes

**DOI:** 10.3390/nu18203307

**Published:** 2026-10-09

**Authors:** Paulina Baran, Katarzyna Szczepanik, Łukasz Kapica, Piotr Mamcarz

**Affiliations:** 1 Department of Psychophysiological Measurements and Human Factor Research, Military Institute of Aviation Medicine, 01-755 Warsaw, Poland; 2 Independent Researcher, 33-100 Tarnow, Poland; 3 Department of Ergonomics, Central Institute for Labour Protection—National Research Institute, 00-701 Warsaw, Poland; lukap@ciop.pl; 4 Institute of Psychology, The John Paul II Catholic University of Lublin, 20-950 Lublin, Poland; piotr.mamcarz@kul.pl

**Keywords:** judo athletes, stress, coping strategies, weight pressure, adolescents, eating behaviours, social support, combat sports

## Abstract

Background: Young athletes in weight-category sports such as judo may experience pressure related to body weight, which can be associated with unhealthy eating. Building on two earlier studies of young Polish judo athletes, this study examined whether ways of coping with stress, weight-related pressure from the social environment, and perceived availability of nutritional support are associated with abnormal eating behaviours. Methods: In this cross-sectional study, 142 judo athletes aged 12–17 years (48 girls, 94 boys) completed questionnaires assessing eating behaviours (the Test of Eating Situation Style and the Three-Factor Eating Questionnaire), coping with stress (the How Do You Cope? inventory, JSR), and a study-specific questionnaire on weight-related pressure and perceived availability of nutritional support. Results: Girls reported higher emotion-focused coping in several sex-by-age subgroup comparisons. After accounting for sex, age, and BMI-for-age z-score, coping styles and weight-related pressure were significantly associated with dietary restraint and emotional eating. Critical comments about an athlete’s weight or body shape and pressure to maintain a specific competition weight were among the most consistent the strongest predictors, whereas BMI-for-age z-score was not. Athletes who perceived broadly defined nutritional support as available reported less eating in response to external cues than those without such support. Conclusions: In this sample of young Polish judo athletes, weight-related comments and pressure, together with coping strategies, are associated with abnormal eating behaviours. Careful coach communication about weight, evaluation of structured nutritional-support provision, and coping-skills training are candidate prevention targets that should be tested in longitudinal and intervention studies.

## 1. Introduction

Adolescence is a developmental period marked by heightened vulnerability to disordered eating, associated with rapid biological, cognitive, and psychosocial change [1,2,3,4]. Diagnostic ambiguity further complicates the picture. Screening instruments validated for adult populations do not always transfer cleanly to adolescent or population-specific samples, underscoring the need for context-sensitive assessment [5]. For young athletes, these normative pressures intersect with the evaluative demands of competitive sport [6,7]. Moreover, meta-analytic evidence indicates that differences in eating-disorder psychopathology between female athletes and non-athletes depend on sport type [8]. On average, female athletes and non-athletes reported similar levels of overall eating-disorder psychopathology, and athletes reported lower body dissatisfaction; however, athletes in aesthetic/lean and weight-category sports, such as judo, showed higher levels of disordered eating than those in sports that do not emphasise leanness [8]. At the same time, competitive sport may also provide resources, such as social support and opportunities to develop coping skills and resilience, that could protect young athletes against these pressures [6]. Whether young athletes are more vulnerable or, conversely, better protected than their non-athlete peers therefore remains an open question [7].

Within this broader context of athletic pressures, judo, as a weight-category combat sport, imposes additional, sport-specific stressors related to body mass management [9,10]. Rapid weight loss practices, in particular, have been reported in the vast majority of practitioners, with weight-related pressures and abnormal eating patterns frequently emerging during critical developmental periods between the ages of 13 and 16 [9,11]. Beyond weight management demands, judo training and competition are associated with measurable psychophysiological stress responses. These have been documented through cortisol and cardiovascular indices during competitive events [12] and through heart-rate variability during precompetitive periods in high-level athletes [13]. Mental stress before competition has long been recognised as a factor requiring active detection and management in this sport [14]. Applied approaches such as EEG biofeedback training, for example, have been explored as tools to support athletes’ self-regulation under competitive pressure [15]. Beyond the competitive setting, judo participation has also been linked to broader psychological correlates in young practitioners, including self-esteem and social positioning within peer groups [16], motivational profiles associated with sustained participation [17], and, in some populations, symptoms of depression among male combat-sport participants [18].

Against this broader psychological backdrop, two companion studies conducted so far in judo population have begun to map eating-related vulnerability specifically. The first established pronounced sex differences in emotional eating among young Polish judo athletes, alongside evidence of divergent psychological responses to comparable sport-related pressures across sexes. It concluded that prevention efforts should specifically target emotion regulation [11]. The second showed that training-related characteristics carried limited predictive value for eating behaviours, whereas relative body mass classification and appearance satisfaction were more consistently associated with dietary restraint [19]. However, this finding suggests that relative body mass and body image alone do not fully account for the eating-related vulnerability observed in this population.

These findings raise an open question, i.e., if body mass and training intensity are not the main correlates of abnormal eating in this population, which psychosocial factors are? One relevant theoretical framework is offered by the transactional model of stress, which proposes that distress arises when perceived environmental demands exceed an individual’s coping resources [7,20]. Competitiveness itself, in turn, has been theorised as a source of chronic stress exposure with downstream psychological consequences [21]. In this context, coping strategies among young athletes in team and individual sports have been shown to vary considerably, with some strategies proving more adaptive than others under competitive pressure [22]. In judo populations specifically, psychological functioning has been linked directly to athletes’ use of sport-coping skills [23]. Pre-start stress coping has similarly been examined among judo competitors, highlighting the range of strategies athletes employ before competition [24].

Beyond coping strategies themselves, in weight-sensitive disciplines, psychosocial demands are frequently expressed through social pressure regarding weight and appearance, including critical comments from coaches, parents, or peers [25,26]. Such pressure may in turn prompt the use of food as a maladaptive affect-regulation strategy [27]. Conversely, the coach–athlete relationship and broader perceived social support have been proposed as protective buffers against psychological difficulties in judo, including through their association with psychological safety and well-being [28]. More broadly, resilience profiles among elite athletes have been linked to a range of health-related behaviours and outcomes [29]. Nutritional knowledge, attitudes, and practices among young athletes have also been identified as a relevant contextual factor for eating-related risk [30]. Relatedly, health-related behaviours more broadly have been documented among women practising judo and ju-jitsu [31]. Support-seeking during help-seeking processes has additionally been studied among student-athlete populations, pointing to systemic and attitudinal barriers that may also be relevant to younger competitive athletes [32]. Weight-related psychosocial pressure therefore represents a second, equally relevant set of candidate predictors alongside coping strategies, and both were examined in the present study.

To our knowledge, no study has examined whether the coping strategies athletes habitually employ under weight-related pressure are associated with the severity of abnormal eating behaviours in judo athletes. Unlike the previous companion studies, which examined sex differences in eating behaviours [11] and the role of training characteristics, relative body mass, and body image [19], the present study, conducted in an independent cohort, focuses on psychosocial stress-coping factors and weight-related psychosocial pressure, assessed here for the first time in this specific judo-athlete cohort using the JSR (Polish: Jak Sobie Radzisz?—How Do You Cope?) inventory. Specifically, we examine (1) whether coping styles differ by sex and age group; (2) whether coping styles and weight-related psychosocial pressure predict eating behaviours beyond sex, age, and BMI; and (3) whether perceived access to nutritional support is associated with the severity of these behaviours. Addressing these questions extends the emotion-regulation-focused prevention target identified in the first companion study [11] by testing specific, modifiable coping strategies and environmental pressures as candidate predictors.

For clarity, the key terms are used in this manuscript as follows. “Abnormal eating behaviours” is an umbrella term for the eating-behaviour outcomes assessed here with screening questionnaires, i.e., dietary restraint, uncontrolled eating, emotional eating, and external eating; it does not denote clinically diagnosed eating disorders. Terms such as “disordered eating” or “eating-disorder psychopathology” are used only when referring to the cited literature. “Coping strategies” (or coping styles) refer to the ways of dealing with stress measured with the JSR inventory, i.e., active problem-focused coping, emotion-focused coping, and support-seeking, whereas “emotion regulation” refers to the broader capacity to manage emotional states, as in the first companion study [11]. “Weight-related psychosocial pressure” refers to critical comments on body weight or shape, perceived pressure to maintain a competition weight, and pre-competition weight-related stress, and “nutritional support” refers to perceived access to nutritional guidance from a dietitian, coach, parents, or psychologist.

## 2. Materials and Methods

### 2.1. Participants and Procedure

Unlike the two previous companion studies, the present analyses were conducted on an independent sample of young Polish judo athletes (*N* = 142; 48 girls, 94 boys; aged 12–17 years, M = 14.4, SD = 1.70; BMI: M = 20.57, SD = 2.83). BMI was calculated as body mass in kilograms divided by height in metres squared (kg/m^2^), based on self-reported height and body mass provided in the questionnaire. Participants had trained judo for an average of 7.62 years (SD = 2.65), reported an average of 4.32 judo training sessions per week (SD = 1.15), and had a mean single training session duration of 99.10 min (SD = 25.50). In addition, 61 athletes (43.0%) reported regularly practising other sports besides judo, and almost all participants reported competing in judo competitions (*N* = 141, 99.3%). This new cohort was recruited specifically to enable the assessment of coping strategies using the JSR inventory, which had not been included in the instrument battery of the earlier studies [11,19].

The study was conducted between February and May 2026, during a training camp for the Małopolska macroregion judo squad, with additional participants recruited from clubs in Wrocław (Juvenia), Opole (AZS Judo), the Wielkopolskie region near Poznań (KS Judo Kuszka), and Jasło (ASW Judo). A convenience sampling method was applied, i.e., athletes who met the inclusion criteria and voluntarily agreed to participate were enrolled in the study. Inclusion criteria were age between 12 and 17 years, active participation in judo training, good general health allowing participation in a questionnaire-based study, and parental/legal guardian consent together with the athlete’s own assent to participate. Exclusion criteria were lack of active judo training (fewer than two training sessions per week), age below 12 or above 17 years, absence of parental/legal guardian or athlete consent, and inability to complete the questionnaires independently due to cognitive, health-related, or language limitations. Written informed consent was obtained both from the athletes themselves and from their parents or legal guardians. The study was conducted in accordance with the Declaration of Helsinki and approved by the Bioethics Committee of the University of Applied Sciences in Tarnow (Decision No. 29/2025).

### 2.2. Measures

Coping strategies were assessed using the JSR (How Do You Cope?) inventory [33]. The instrument distinguishes between dispositional (habitual) and situational (context-specific) coping. Each of these two domains comprises nine items measuring three specific dimensions: active problem-focused coping, emotion-focused coping, and support-seeking, resulting in six subscales in total (three items per subscale). Participants rate the frequency (dispositional part) or intensity (situational part) of each behaviour on a five-point Likert scale (dispositional part: 0 = almost never to 4 = almost always; situational part: 0 = definitely not to 4 = definitely yes). Example items include “I’m trying to think carefully about what I can do in this situation” and “I keep myself busy with something else, just to take my mind off my troubles”. Subscale scores are calculated as the mean of the respective items (range: 0–4), with higher scores indicating more frequent use of a given coping strategy. The JSR has demonstrated robust reliability and validity within the Polish population of children and adolescents [33]. In the present study, McDonald’s omega coefficients for the subscales ranged from 0.67 to 0.82.

Eating behaviours were assessed with the same two instruments used in the companion studies to preserve comparability of outcome measures across the series. The Test of Eating Situation Style (TSJ) [34] is a Polish-validated instrument comprising nine items describing eating in response to specific emotional and social circumstances, rated on a three-point frequency scale. It yields two subscales: emotional eating (five items) and eating in response to external stimuli (four items). Example items include “I eat when I’m sad” and “I eat while watching TV”. Responses are scored as 1 = rarely, 2 = sometimes, 3 = often. In the present analyses, TSJ subscale scores were expressed as item means rather than raw sums (range: 1–3), with higher scores indicating more frequent eating in response to emotional states or external stimuli, respectively. In the present study, McDonald’s omega coefficients were 0.90 for emotional eating and 0.65 for eating in response to external stimuli. The Three-Factor Eating Questionnaire, short form (TFEQ-13 [35]; Polish adaptation [36]), captures three dimensions of eating behaviour—dietary restraint (conscious restriction of food intake to control body weight), uncontrolled eating (a tendency to lose control over eating and eat more than usual), and emotional eating (overeating in response to negative emotional states)—through 13 items on a predominantly four-point response format. Dietary restraint and uncontrolled eating are each measured with five items, and emotional eating with three items. Example items include “I’m always hungry, and that’s why I find it hard to stop eating until I’ve finished everything on my plate” and “I consciously control the amount of food I eat at mealtimes so as not to put on weight”. Responses to the four-point items are scored from 0 (definitely not) to 3 (definitely yes); the single item answered on an eight-point scale is recoded to a 0–3 range. In the present analyses, TFEQ-13 subscale scores were also expressed as item means rather than raw sums (range: 0–3); no total score was computed. Higher scores indicate greater dietary restraint, uncontrolled eating, or emotional eating, respectively. In the present study, McDonald’s omega coefficients were 0.85 for dietary restraint, 0.73 for uncontrolled eating, and 0.88 for emotional eating. As the JSR is a copyrighted instrument and the TSJ was made available for research purposes with the permission of its author, only selected example items (translated by the authors) are quoted, and the full wording of the instruments is not reproduced.

Psychosocial weight-related pressure and perceived access to nutritional support were assessed using six items (Q14–Q19) drawn from a broader, study-specific 19-item questionnaire developed for the present research programme. The remaining 13 items of this questionnaire captured sociodemographic, anthropometric, and training-related characteristics and were used for sample description in the present study (Section 2.1); they were not entered as predictors in the present analyses. The six items used here were: (Q14) whether the athlete’s weight or body shape had been commented on by others, and by whom (no one; yes, coach; yes, parents; yes, teammates; yes, other people); (Q15) the frequency of perceived pressure to maintain a specific competition weight (five-point scale: never to very often); (Q16) the perceived source of that pressure (self, coach, parents, or club teammates), collected in the questionnaire but not included in the regression models because it was a conditional multiple-response item rather than a single ordinal predictor; (Q17) pre-competition weight-related stress (five-point scale: not at all to a lot, or not applicable for non-competing athletes); (Q18) the timing of weight-control practices relative to competition (four categories: no control, a few days before, one week before, or two weeks or more before); and (Q19) perceived access to nutritional support (dietitian, coach, parents, or psychologist), coded as a three-level categorical variable (available; unavailable; desired but unavailable). The questionnaire was developed by the authors specifically for the purposes of the present study; it was neither pilot-tested nor formally validated. Its items were designed as brief, direct questions about specific experiences (e.g., whether weight-related comments had occurred or how often pressure was perceived) and were therefore analysed as single-item indicators rather than as a psychometric scale.

### 2.3. Statistical Analyses

Statistical analyses were conducted using R statistical software (version 4.3.3; R Foundation for Statistical Computing, Vienna, Austria). Descriptive statistics were calculated for all study variables.

Sex differences in JSR coping subscales were examined separately within two age groups (12–14 and 15–17 years) using Welch’s independent-samples *t*-tests, which do not assume equal variances across groups. This two-group age split was adopted to obtain more comparable subgroup sizes for these analyses. Hedges’ g was used as the effect size measure. To account for multiple testing across the 12 sex comparisons (six JSR subscales × two age groups), *p*-values were additionally adjusted using the Benjamini–Hochberg procedure. Both unadjusted and adjusted *p*-values are reported, whereas statistical interpretation was based on the adjusted *p*-values.

Given the adolescent age range of the sample, BMI-for-age z-scores were calculated from the self-reported height and body mass data described above using the WHO 2007 age- and sex-specific reference values for school-aged children and adolescents, implemented in the R package *anthroplus* [37,38,39]. BMI-for-age z-score was used as the anthropometric covariate in the hierarchical regression models.

Hierarchical multiple regression analyses were conducted to examine the incremental predictive value of coping styles and weight-related psychosocial variables in explaining eating behaviours. Five eating-behaviour outcomes were analysed: TFEQ dietary restraint, TFEQ uncontrolled eating, TFEQ emotional eating, TSJ emotional eating, and TSJ external eating. In all models, sex, age, and BMI-for-age z-score were entered in Block 1, the six JSR coping subscales were entered in Block 2, and five study-specific weight-related psychosocial predictors were entered in Block 3: the presence of comments about the athlete’s weight or body shape, the frequency of perceived pressure to maintain a specific competition weight, pre-competition weight-related stress, the timing of weight-control practices relative to competition, and perceived access to nutritional support. Nutritional support was treated as a three-level categorical predictor, with no support as the reference category; thus, two contrasts were estimated: desired but unavailable support vs. no support, and available support vs. no support. The single non-competing participant was retained in analyses for which the relevant variables were applicable; however, because the pre-competition weight-related stress item referred specifically to stress experienced before judo competitions, this participant’s response was treated as not applicable and coded as missing for this variable. The perceived source of weight-maintenance pressure was assessed in the questionnaire but was not included in the regression models because it was a conditional multiple-response item rather than a single ordinal or categorical predictor suitable for direct inclusion. For each outcome, three nested linear regression models were estimated and compared sequentially. R^2^ was used to quantify the cumulative proportion of variance explained by the predictors entered up to a given block, whereas ΔR^2^ was calculated as the difference in R^2^ between two consecutive nested models. The statistical significance of each ΔR^2^ was evaluated using the corresponding F-change test. Adjusted R^2^ was reported for the final model to account for the number of predictors included. In the final models, unstandardised regression coefficients (B), 95% confidence intervals for B, standardised coefficients (β), and *p*-values were reported for statistically significant predictors. The hierarchical regression analyses were conducted using complete cases for the full set of predictors included in the final models.

Differences in eating behaviours across the three nutritional-support groups (no support, desired but unavailable support, and available support) were examined using Welch’s ANOVA. Post hoc pairwise comparisons were conducted only for outcomes with a statistically significant omnibus test and were corrected using the Benjamini–Hochberg procedure. Effect sizes are reported as Hedges’ g for pairwise comparisons and η^2^ for omnibus group comparisons. Statistical significance was set at *p* < 0.05.

## 3. Results

### 3.1. Sex and Age Differences in Coping Styles

Descriptive and distributional characteristics of the main continuous and ordinal study variables are provided in Table A1 in Appendix A. Independent-samples *t*-tests comparing JSR coping subscales between girls and boys, conducted separately within each age group (12–14 and 15–17 years), identified four statistically significant sex differences, summarised in Table 1.

**Figure 1:**
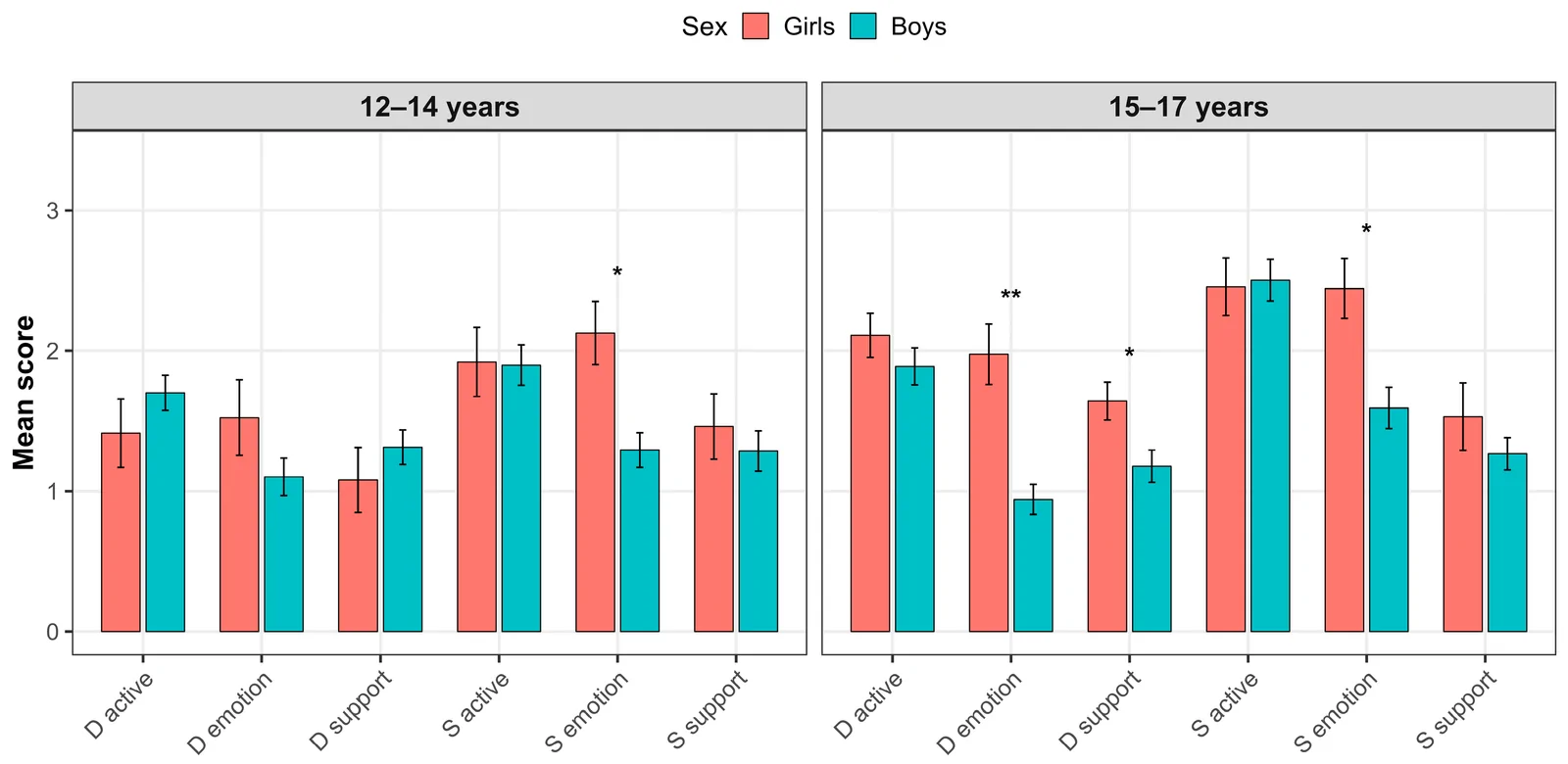
JSR coping styles by sex and age group (12–14 and 15–17 years). Asterisks indicate statistically significant sex differences from Table 1 (no additional tests performed). Note: * *p*_BH_ < 0.05; ** *p*_BH_ < 0.01; D—Dispositional; S–Situational.

According to the data in Table 1, girls reported significantly higher situational emotion-focused coping than boys in both age groups. In the 15–17-year age group, girls also reported higher dispositional emotion-focused coping and dispositional support-seeking than boys. These subgroup comparisons are descriptive and should not be interpreted as evidence of a formal sex-by-age interaction. As a descriptive visual summary of Table 1, mean scores for all six JSR subscales by sex and age group are presented in Figure 1.

Additional sex comparisons for the TFEQ and TSJ eating-behaviour outcomes within each age group are reported in Table A2 in Appendix A. After Benjamini–Hochberg correction, within the 15–17-year age group, girls reported higher TFEQ dietary restraint and TFEQ emotional eating than boys.

### 3.2. Coping Styles and Weight-Related Pressure as Predictors of Eating Behaviours

Model summaries for the hierarchical regression analyses, testing the incremental contribution of JSR coping subscales (Block 2) and weight-related psychosocial pressure and perceived nutritional support (Block 3) beyond sex, age, and BMI z-score (Block 1), are presented in Table 2 for each of the five eating-behaviour outcomes. In these models, each block was evaluated by the increase in explained variance relative to the previous block, with R^2^ indicating cumulative explained variance and ΔR^2^ indicating the additional variance explained by the newly added predictors.

Notably, Block 3 comprised five of the six study-specific items described in Section 2.2—whether the athlete’s weight/shape had been the subject of critical comments (and by whom), the frequency of perceived weight-maintenance pressure, pre-competition weight-related stress, the timing of weight-control practices relative to competition, and perceived access to nutritional support. The perceived source of that pressure (Q16) was not entered as a separate predictor because it was a conditional multiple-response item rather than a single ordinal or categorical variable suitable for direct inclusion in the regression models.

R^2^ values in Table 2 represent the cumulative proportion of variance explained by the predictors entered up to a given block, whereas ΔR^2^ indicates the additional variance explained by the predictors newly added at each step. For TFEQ dietary restraint, the control variables entered in Block 1 explained 5.1% of the variance. Adding the JSR coping subscales in Block 2 explained a further 12.6 percentage points (ΔR^2^ = 0.126, *p* = 0.005), resulting in a cumulative R^2^ of 0.177. Adding weight-related pressure and nutritional-support variables in Block 3 explained a further 23.3 percentage points (ΔR^2^ = 0.233, *p* < 0.001). The final model explained 41.1% of the variance (R^2^ = 0.411; adjusted R^2^ = 0.339).

For TFEQ uncontrolled eating, Block 1 explained 17.2% of the variance. The addition of JSR coping subscales in Block 2 increased explained variance by 6.0 percentage points, but this increment was not statistically significant (ΔR^2^ = 0.060, *p* = 0.128). The addition of weight-related pressure and nutritional-support variables in Block 3 explained a further 5.5 percentage points, which was also not statistically significant (ΔR^2^ = 0.055, *p* = 0.161). The final model explained 28.7% of the variance (R^2^ = 0.287; adjusted R^2^ = 0.200).

For TFEQ emotional eating, Block 1 explained 11.7% of the variance. Adding JSR coping subscales in Block 2 explained a further 25.1 percentage points (ΔR^2^ = 0.251, *p* < 0.001), and adding weight-related pressure and nutritional-support variables in Block 3 explained an additional 10.7 percentage points (ΔR^2^ = 0.107, *p* = 0.001). The final model explained 47.4% of the variance (R^2^ = 0.474; adjusted R^2^ = 0.410).

For TSJ emotional eating, Block 1 explained 7.5% of the variance. Neither the addition of JSR coping subscales in Block 2 (ΔR^2^ = 0.060, *p* = 0.187) nor the addition of weight-related pressure and nutritional-support variables in Block 3 (ΔR^2^ = 0.069, *p* = 0.107) produced a statistically significant increase in explained variance. The final model explained 20.4% of the variance (R^2^ = 0.204; adjusted R^2^ = 0.107).

For TSJ external eating, Block 1 explained 2.9% of the variance. Adding JSR coping subscales in Block 2 explained a further 13.2 percentage points (ΔR^2^ = 0.132, *p* = 0.004), resulting in a cumulative R^2^ of 0.161. Adding weight-related pressure and nutritional-support variables in Block 3 explained an additional 7.5 percentage points, although this increment did not reach conventional statistical significance (ΔR^2^ = 0.075, *p* = 0.070). The final model explained 23.5% of the variance (R^2^ = 0.235; adjusted R^2^ = 0.142).

Statistically significant predictors from the final (Block 3) models across all five outcomes are summarised in Table 3.

As shown in Table 3, BMI z-score showed no significant unique association with any outcome across the five final models, and sex was similarly non-significant once coping styles and weight-related and nutritional-support variables were entered. Critical comments on weight or body shape emerged as the most consistent predictor, reaching significance for three of the five outcomes, i.e., TFEQ dietary restraint, TFEQ emotional eating, and TSJ emotional eating. It is worth noting that for TSJ emotional eating specifically, the Block 3 increment in explained variance was not statistically significant (ΔR^2^ = 0.069, *p* = 0.107; Table 2), even though critical comments on weight/shape emerged as a significant individual predictor within that block. Such a pattern—a significant single coefficient alongside a non-significant omnibus block test—can arise when the remaining block predictors contribute little unique variance; this result should therefore be treated as more tentative than the other Block 3 effects, pending replication.

For TFEQ dietary restraint, significant individual predictors included critical comments on weight or body shape, perceived weight-maintenance pressure, timing of weight control, and perceived nutritional support desired but unavailable compared with no support. Perceived weight-maintenance pressure was additionally a significant predictor of both TFEQ dietary restraint and TFEQ emotional eating, but not of TSJ emotional eating. No weight-related pressure or support variable reached significance for TFEQ uncontrolled eating, where age was the sole significant predictor. For TSJ external eating, the significant predictors were instead JSR dispositional support-seeking coping, positively associated, and perceived availability of nutritional support, negatively associated, discussed further in Section 3.3.

Regarding coping styles specifically, TFEQ emotional eating was positively associated with dispositional emotion-focused coping and dispositional support-seeking coping, but negatively associated with situational support-seeking coping. TSJ external eating was positively associated with dispositional support-seeking coping. This mixed pattern of associations involving coping styles is discussed below.

### 3.3. Nutritional Support and Eating Behaviours

Of the 142 athletes, 60 reported no access to nutritional support, 18 reported wanting but lacking such support, and 64 reported having support available. Differences in eating behaviours across these three nutritional-support groups, tested using Welch’s ANOVA, are presented in Table 4 (significant comparisons only).

Post hoc comparisons (Benjamini–Hochberg corrected) indicated that athletes with available support reported significantly lower external eating than those without support (*p* = 0.012, g = −0.527). The same pattern held relative to those desiring but lacking support (*p* = 0.049, g = −0.551). For uncontrolled eating, the omnibus effect was significant, but none of the pairwise comparisons reached statistical significance after correction; the difference between athletes with available support and those without support was the closest to significance (*p* = 0.051, g = −0.434). No significant group differences emerged for dietary restraint or TFEQ/TSJ emotional eating (all *p* > 0.07).

## 4. Discussion

The present findings extend the companion series [11,19] by identifying specific psychosocial factors associated with abnormal eating behaviours in young judo athletes, independently of relative body mass and body image. Prior studies in this population of judo competitors documented pronounced sex differences in emotional eating and supported the need for prevention strategies targeting emotion regulation [11]. Consistent with this, the present results show that girls—particularly older adolescents—more frequently rely on emotion-focused coping strategies, both dispositionally and situationally. This pattern is broadly consistent with evidence that coping strategies among young athletes vary in their adaptiveness under competitive stress [22], and with judo-specific evidence linking psychological functioning to the coping skills athletes employ [23] and to strategies used specifically around competition [24].

Although the cross-sectional design does not allow causal conclusions, these associations are compatible with the transactional model of stress, according to which abnormal eating may emerge when perceived weight-related demands exceed available adaptive coping resources [7,20]. This interpretation is compatible with broader evidence that chronic exposure to competitive pressure carries psychological costs [21]. ^I^t is also consistent with documented psychophysiological stress responses among judo athletes around competition, including elevated physiological arousal [12,13]. Accordingly, structured stress detection and management have long been recognised as necessary in this sport [14]. Self-regulatory training approaches, such as EEG biofeedback, have been explored as a means of supporting athletes’ stress management capacity [15], and may represent a complementary avenue alongside psychosocial interventions targeting coping style directly.

Critically, weight-related psychosocial pressure—particularly critical comments about weight or body shape from coaches, parents, or peers, and perceived pressure to maintain a specific competition weight—emerged as the strongest predictors of dietary restraint and emotional eating, explaining substantial variance beyond sex, age, and BMI. Notably, the relative contribution of the two sets of predictors differed by outcome: for dietary restraint, weight-related variables explained more additional variance than coping styles (ΔR^2^ = 0.233 vs. 0.126), whereas for TFEQ emotional eating the reverse was observed (ΔR^2^ = 0.107 vs. 0.251; Table 2). Weight-related psychosocial factors therefore deserve at least as much attention as coping styles when interpreting eating behaviours in this population. This finding complements the second companion study, where relative weight classification and appearance satisfaction, rather than training intensity, were the more consistent correlates of restrictive eating [19]. The present results extend this picture, i.e., the psychosocial interpretation of weight-related demands, rather than relative body mass and body image themselves, may be more proximally associated with these behaviours. Critical weight-related comments from the athlete’s social microsystem align with evidence that such commentary can distort body perception and trigger restrictive or emotionally driven eating cycles [25,26], and are broadly consistent with meta-analytic evidence that eating-disorder risk in athletic populations can diverge from that of non-athletes in sport-specific ways [8].

Beyond weight-related pressure itself, the pattern of coping-style associations also warrants attention. TFEQ emotional eating was positively associated with dispositional emotion-focused coping and dispositional support-seeking coping, but negatively associated with situational support-seeking coping. This is consistent with the proposed protective role of social support and high-quality relational contexts in buffering athletes against maladaptive affect regulation [28], and with broader evidence linking athlete resilience profiles to more favourable health-related outcomes [29]. Dispositional support-seeking coping was also positively associated with TFEQ emotional eating, suggesting that dispositional and situational forms of support-seeking may capture different aspects of coping in this context. Conversely, dispositional support-seeking coping was positively associated with external eating (TSJ)—a divergent pattern that may reflect differences between habitual reliance on others as a coping resource and the situational activation of support-seeking under acute stress. Hence, this distinction merits further investigation, potentially informed by broader work on help-seeking behaviour among young athletes, which has identified systemic and attitudinal barriers to accessing support [32].

The favourable association between perceived access to nutritional support and lower external eating adds a practically relevant, potentially modifiable factor to this picture. Notably, coaches occupy a particularly influential role in young athletes’ weight-related decisions [26]. Formal nutritional guidance may therefore be associated with a lower risk of maladaptive eating patterns associated with weight-category sport participation, complementing calls for structured assessments of knowledge, attitudes, and practices to inform evidence-based nutrition and health education tailored to young athletes [30]. This is consistent with broader documentation of health-related behaviours among female judo and ju-jitsu practitioners [31]. Many young athletes begin weight-cutting practices without professional guidance, relying instead on extreme caloric restriction, which is associated with irritability, tension, and reduced motivation [9,10]; the present findings suggest that structured nutritional support may be linked to lower risk in this respect. Programmes addressing disordered eating in athletic settings should also be considered against the broader backdrop of the clinical spectrum of eating disorders documented across sport more generally [27]. Relatedly, it is worth noting that competitive and recreational judokas are generally characterised by high self-esteem and positive social standing [16], and these psychosocial resources and associated peer dynamics may be related to how young judo athletes experience and respond to these pressures, as may their underlying motivational engagement with the sport [17].

Finally, the present study did not assess depressive symptoms directly. However, prior evidence of psychological difficulties, including depressive symptomatology, among young combat-sport participants [18] underscores that the eating-related vulnerability documented here should be considered within a broader mental health context rather than in isolation. Overall, this echoes calls for developmentally sensitive, diagnostically informed screening approaches in adolescent populations [1,4,5].

Taken together with the two companion studies, the present results point toward a layered picture of vulnerability in this population. First, relative body mass and appearance dissatisfaction are associated with susceptibility to restraint [19]. Second, sex differences in emotional eating may reflect divergent affective responses to shared sport pressures [11]. And finally, as shown here, perceived psychosocial pressure and habitual coping strategies are further associated with the severity and form of these behaviours, independently of relative body mass and body image.

### 4.1. Limitations

Nevertheless, several limitations warrant consideration. First, the cross-sectional design precludes causal inference. The observed associations between coping style, psychosocial pressure, and eating behaviours therefore cannot establish directionality or rule out reverse or bidirectional relationships. Second, all measures relied on self-report, which may introduce response bias, particularly for sensitive topics such as weight-related comments and eating behaviours, although validated instruments were used for the psychometric outcomes. Additionally, BMI and BMI-for-age z-scores were calculated from self-reported height and body mass. This should be interpreted with caution, particularly in a weight-category sport such as judo, where body mass may be a sensitive and strategically managed variable. Future studies should include objective anthropometric assessment and, where possible, more detailed indicators of body composition. In contrast, weight-related pressure and perceived access to nutritional support were assessed with single items from a study-specific questionnaire that was neither pilot-tested nor formally validated; their psychometric properties should therefore be established in future research. Notably, the TSJ external-eating subscale showed relatively modest internal consistency (ω = 0.65); however, lower reliability tends to attenuate rather than inflate observed associations, suggesting the corresponding finding is unlikely to be an artefact of measurement error, though replication with a more reliable measure is warranted. Third, subgroup sizes in the sex × age-group comparisons of coping styles were modest, particularly for girls (*N* = 21–27 per age group, versus *N* = 45–49 for boys), limiting statistical power to detect smaller effects and warranting caution in generalising these specific comparisons. Fourth, given the number of predictors tested across five regression models (six JSR subscales plus five study-specific weight-related/support predictors per outcome, with perceived nutritional support represented by two dummy-coded contrasts), no formal correction for multiple comparisons was applied to the regression coefficients. Results should therefore be interpreted with appropriate caution, and findings—particularly those at borderline significance—should be considered hypothesis-generating pending replication. Fifth, as with the two companion studies, the sample was drawn from selected judo clubs across Poland using a convenience sampling method, in which participation depended on athletes voluntarily agreeing to take part. This approach may have introduced self-selection bias—for instance, athletes more engaged with or affected by weight-related pressure may have been more or less likely to participate. Moreover, together with the reliance on selected clubs, it may limit generalisability to athletes training in different regional or organisational contexts, as well as to other populations and sporting contexts. Sixth, the present cohort is independent from that examined in the first companion study, and coping styles here were analysed using a two-group age split (12–14 vs. 15–17 years). Direct comparisons of age-specific patterns across the two studies should therefore be made with caution. Seventh, the study did not include a non-athlete comparison group; therefore, it cannot be determined whether young judo athletes are more vulnerable to abnormal eating than their non-athlete peers or whether sport participation also provides protective resources. Future studies should include matched non-athlete controls.

### 4.2. Practical Implications

These findings carry several implications for coaches, sports medicine professionals, and those responsible for athlete welfare in youth judo settings. First, critical comments about weight or body shape emerged as among the most consistent predictors of both dietary restraint and emotional eating. Coach and parent education focused on the potential harm of weight-related commentary—even when well-intentioned—therefore represents a candidate target for future intervention research [25,26]. Second, the favourable association between perceived access to nutritional support and lower external eating suggests that making structured, professionally guided nutritional support more consistently available to young athletes is worth evaluating. This support should not be left to informal or self-directed weight management practices [9,30]. Third, the elevated reliance on emotion-focused coping among older girls, combined with its association with emotional eating, points to a specific, modifiable target for prevention programming: structured training in adaptive coping and emotion regulation skills, particularly for girls approaching mid-to-late adolescence, which may also help foster supportive networks and psychological safety associated with mental well-being in judo [28]. Fourth, the favourable pattern observed for situational support-seeking (as opposed to dispositional support-seeking) suggests that interventions might usefully focus on helping athletes activate support-seeking behaviour specifically in moments of acute weight-related stress, rather than assuming that general reliance on others is uniformly protective. Finally, coaches are frequently the most influential figures in young athletes’ weight-related decisions [26]. Equipping them with both the knowledge to recognise problematic eating behaviours and the communication skills to avoid reinforcing weight-related pressure may therefore represent one of the most direct and scalable prevention avenues available in this setting.

## 5. Conclusions

This study identified psychosocial pressure related to weight—particularly critical comments about weight or body shape and perceived pressure to maintain a specific competition weight—as consistently associated with dietary restraint and emotional eating among young Polish judo athletes, independently of sex, age, and BMI-for-age z-score calculated using age- and sex-specific reference values. Given the cross-sectional design, these findings reflect associations rather than causal effects. Coping styles further contributed to this picture. Girls reported higher emotion-focused coping in several subgroup comparisons, although age-specific patterns should be interpreted cautiously. Emotion-focused coping was itself associated with elevated emotional eating. Situational support-seeking, by contrast, showed an inverse, potentially protective association. Perceived availability of broadly defined nutritional support was associated with lower external eating, suggesting it as a further potentially modifiable factor to be evaluated in longitudinal and intervention studies. Considered alongside the two companion investigations in this series, these findings suggest that abnormal eating behaviours among young Polish judo athletes are associated not only with relative body mass, body image, and training demands, but also—independently— with the psychosocial climate surrounding weight, i.e., particularly critical commentary and perceived pressure. They are also related to the coping resources athletes have available to manage this pressure. Prevention efforts in this population should be evaluated through future longitudinal and intervention research combining coach and parent education on weight-related communication, evaluation of structured nutritional-support provision, and targeted coping-skills training for adolescent athletes, especially girls. As the sample was recruited by convenience sampling from selected Polish judo clubs, these conclusions are restricted to the population examined and should not be generalised to other populations or sporting contexts without further research.

## Figures and Tables

**Table 1:** Coping styles (JSR) by sex and age group.

Age Group	Scale	Girls *n*	Girls M (SD)	Boys *n*	Boys M (SD)	t	*df*	*p*	*p*_BH_	Hedges’ g
12–14	Dispositional—active problem-focused	21	1.41 (1.12)	49	1.70 (0.87)	−1.05	30.9	0.300	0.493	−0.30
	Dispositional—emotion-focused	21	1.52 (1.23)	49	1.10 (0.93)	1.41	30.2	0.170	0.408	0.41
	Dispositional—support-seeking	21	1.08 (1.06)	49	1.31 (0.86)	−0.89	31.6	0.380	0.507	−0.25
	Situational—active problem-focused	21	1.92 (1.12)	49	1.90 (1.01)	0.08	34.3	0.937	0.937	0.02
	Situational—emotion-focused	21	2.13 (1.03)	49	1.29 (0.86)	3.26	32.5	0.003	0.011	0.90
	Situational—support-seeking	21	1.46 (1.07)	49	1.29 (1.00)	0.64	35.8	0.527	0.632	0.17
15–17	Dispositional—active problem-focused	27	2.11 (0.82)	45	1.89 (0.88)	1.09	58.1	0.282	0.493	0.26
	Dispositional—emotion-focused	27	1.98 (1.12)	45	0.94 (0.72)	4.31	39.1	<0.001	0.001	1.15
	Dispositional—support-seeking	27	1.64 (0.70)	45	1.18 (0.77)	2.62	59.4	0.011	0.033	0.62
	Situational—active problem-focused	27	2.46 (1.07)	45	2.50 (1.00)	−0.18	52.2	0.854	0.932	−0.05
	Situational—emotion-focused	27	2.44 (1.11)	45	1.59 (0.99)	3.27	50.0	0.002	0.011	0.81
	Situational—support-seeking	27	1.53 (1.25)	45	1.27 (0.77)	0.99	38.0	0.328	0.493	0.27

*Note.* JSR subscale scores are item-mean scores ranging from 0 to 4. *p*_BH_ denotes Benjamini–Hochberg-adjusted *p*-values across the 12 sex comparisons. Hedges’ g is positive when girls scored higher than boys.

**Table 2:** Hierarchical regression model summaries for five eating-behaviour outcomes (*N* = 141).

Outcome	Block 1 R^2^	Block 2 R^2^ (ΔR^2^, *p*)	Block 3 R^2^ (ΔR^2^, *p*)	Adj. R^2^ (Final)
TFEQ—Dietary Restraint	0.051	0.177 (0.126, 0.005)	0.411 (0.233, <0.001)	0.339
TFEQ—Uncontrolled Eating	0.172	0.232 (0.060, 0.128)	0.287 (0.055, 0.161)	0.200
TFEQ—Emotional Eating	0.117	0.368 (0.251, <0.001)	0.474 (0.107, 0.001)	0.410
TSJ—Emotional Eating	0.075	0.135 (0.060, 0.187)	0.204 (0.069, 0.107)	0.107
TSJ—External Eating	0.029	0.161 (0.132, 0.004)	0.235 (0.075, 0.070)	0.142

*Note.* Block 1: sex, age, BMI z-score. Block 2: +JSR coping subscales. Block 3: +weight-related pressure and perceived access to nutritional support. R^2^ denotes the cumulative proportion of variance explained by the model up to a given block. ΔR^2^ denotes the additional variance explained by the predictors added at that step, relative to the previous block. The hierarchical regression analyses were conducted using complete cases for the full set of predictors included in the final models, resulting in an analytic sample of *N* = 141.

**Table 3:** Statistically significant predictors across all five regression models (*N* = 141).

Outcome	Predictor	B	95% CI for B	β	*p*
TFEQ—Dietary restraint	Comments about weight/body shape	0.286	[0.070, 0.503]	0.402	0.010
TFEQ—Dietary restraint	Weight-related pressure	0.229	[0.113, 0.345]	0.397	<0.001
TFEQ—Dietary restraint	Timing of weight control	0.132	[0.003, 0.262]	0.176	0.046
TFEQ—Dietary restraint	Nutritional support: desired but unavailable vs. none	−0.343	[−0.681, −0.004]	−0.480	0.047
TFEQ—Uncontrolled eating	Age	0.144	[0.083, 0.206]	0.412	<0.001
TFEQ—Emotional eating	Age	0.078	[0.013, 0.143]	0.182	0.018
TFEQ—Emotional eating	JSR dispositional emotion-focused	0.156	[0.015, 0.296]	0.221	0.030
TFEQ—Emotional eating	JSR dispositional support-seeking	0.173	[0.005, 0.341]	0.200	0.044
TFEQ—Emotional eating	JSR situational support-seeking	−0.184	[−0.318, −0.050]	−0.252	0.008
TFEQ—Emotional eating	Comments about weight/body shape	0.334	[0.125, 0.544]	0.458	0.002
TFEQ—Emotional eating	Weight-related pressure	0.136	[0.024, 0.248]	0.230	0.018
TSJ—Emotional eating	Comments about weight/body shape	0.249	[0.053, 0.444]	0.450	0.013
TSJ—External eating	JSR dispositional support-seeking	0.154	[0.034, 0.274]	0.301	0.013
TSJ—External eating	Nutritional support: available vs. none	−0.210	[−0.363, −0.056]	−0.484	0.008

*Note.* B–unstandardised regression coefficient; β—standardised regression coefficient; non-significant predictors (including sex and BMI z score in all five models) were omitted for brevity.

**Table 4:** Eating behaviours by nutritional support group (*N* = 142)—significant comparisons only.

Variable	No Support M (SD)	Desired Support M (SD)	Available Support M (SD)	F (*df*1,*df*2)	*p*	η^2^
TFEQ—Uncontrolled Eating	1.37 (0.60)	1.46 (0.66)	1.12 (0.54)	3.82 (2, 45.9)	0.029	0.053
TSJ—External Eating	2.34 (0.45)	2.33 (0.36)	2.11 (0.41)	5.12 (2, 51.2)	0.009	0.068

*Note.* Values are item-mean scores. TSJ scores range from 1 to 3, and TFEQ−13 scores range from 0 to 3. Only statistically significant comparisons shown.

## Data Availability

The datasets generated during this study are not publicly available due to ethical restrictions and the protection of minors’ privacy, but they are available from the corresponding author upon reasonable request.

## Appendix A

**Table A1:** Descriptive and distributional characteristics of the main study variables.

Variable	*N*	Min	Max	M	SD	Skewness	Kurtosis
Age	142	12.00	17.00	14.40	1.70	0.04	−1.26
BMI	142	14.30	27.40	20.60	2.84	0.35	−0.45
BMI z-score	142	−2.27	2.32	0.20	0.90	−0.06	−0.19
TFEQ—Dietary restraint	142	0.00	3.00	1.28	0.71	0.32	−0.51
TFEQ—Uncontrolled eating	142	0.00	3.00	1.27	0.59	0.16	−0.15
TFEQ—Emotional eating	142	0.00	3.00	0.77	0.72	0.94	0.53
TSJ—Emotional eating	142	1.00	3.00	1.39	0.55	1.60	1.66
TSJ—External eating	142	1.00	3.00	2.23	0.43	−0.22	−0.40
JSR—Dispositional active problem-focused	142	0.00	3.67	1.80	0.92	−0.22	−0.56
JSR—Dispositional emotion-focused	142	0.00	4.00	1.28	1.03	0.64	−0.24
JSR—Dispositional support-seeking	142	0.00	3.33	1.30	0.85	0.20	−0.48
JSR—Situational active problem-focused	142	0.00	4.00	2.20	1.06	−0.48	−0.69
JSR—Situational emotion-focused	142	0.00	4.00	1.73	1.06	0.19	−0.60
JSR—Situational support-seeking	142	0.00	4.00	1.35	0.99	0.43	−0.47
Weight-related pressure	142	0.00	4.00	1.46	1.22	0.60	−0.54
Pre-competition weight-related stress	141	0.00	3.00	1.33	0.94	0.25	−0.83
Timing of weight control	141	0.00	3.00	1.85	0.97	−0.40	−0.85

*Note.* TFEQ, TSJ, and JSR scores are item-mean scores. Raw BMI was calculated as body mass in kilograms divided by height in metres squared (kg/m^2^), based on self-reported height and body mass. BMI-for-age z-scores were calculated using age- and sex-specific reference values. Kurtosis is reported as excess kurtosis. Weight-related pressure, pre-competition weight-related stress, and timing of weight control were ordinal predictors entered in Block 3 of the hierarchical regression models.

**Table A2:** Sex differences in eating-behaviour outcomes by age group.

Age Group	Outcome	Girls *N*	Girls M (SD)	Boys *N*	Boys M (SD)	t	*df*	*p*	*p*_BH_	Hedges’ g
12–14	TFEQ—Dietary restraint	21	1.06 (0.48)	49	1.29 (0.67)	−1.67	51.7	0.101	0.253	−0.38
12–14	TFEQ—Uncontrolled eating	21	1.00 (0.56)	49	1.03 (0.54)	−0.23	36.7	0.822	0.841	−0.06
12–14	TFEQ—Emotional eating	21	0.51 (0.71)	49	0.62 (0.66)	−0.61	35.6	0.545	0.841	−0.16
12–14	TSJ—Emotional eating	21	1.24 (0.31)	49	1.27 (0.48)	−0.37	56.3	0.716	0.841	−0.08
12–14	TSJ—External eating	21	2.19 (0.43)	49	2.17 (0.38)	0.20	34.1	0.841	0.841	0.05
15–17	TFEQ—Dietary restraint	27	1.70 (0.77)	45	1.11 (0.71)	3.24	51.3	0.002	0.010	0.80
15–17	TFEQ—Uncontrolled eating	27	1.56 (0.63)	45	1.49 (0.48)	0.47	43.9	0.639	0.841	0.12
15–17	TFEQ—Emotional eating	27	1.35 (0.87)	45	0.70 (0.51)	3.49	36.9	0.001	0.010	0.95
15–17	TSJ—Emotional eating	27	1.70 (0.64)	45	1.39 (0.59)	2.02	51.3	0.048	0.161	0.50
15–17	TSJ—External eating	27	2.38 (0.39)	45	2.24 (0.50)	1.32	64.7	0.190	0.381	0.30

*Note.* TFEQ and TSJ scores are item-mean scores. TFEQ-13 scores range from 0 to 3, and TSJ scores range from 1 to 3. *p*_BH_ denotes Benjamini–Hochberg-adjusted *p*-values across the 10 sex comparisons. Hedges’ g is positive when girls scored higher than boys.
